# Burnout in the allergy nursing workforce in the United States

**DOI:** 10.3389/frhs.2026.1669523

**Published:** 2026-04-10

**Authors:** Olga Kagan, Lilly Mathew, Hemant Sharma, Sarah Pederson, Ali Doppelt, Anil Nanda, Anita Wasan, Theresa A. Bingemann

**Affiliations:** 1 The Barbara H. Hagan School of Nursing and Health Sciences, Molloy College, Rockville Center, NY, United States; 2 The City University of New York School of Professional Studies, New York, NY, United States; 3 Food Allergy Nursing Association, Great Neck, NY, United States; 4 Northwell Health, Glen Cove, NY, United States; 5 Children's National Hospital, Washington, DC, United States; 6 Department of Allergy and Immunology, Stanford Children Hospital, Palo Alto, CA, United States; 7 Jaffe Food Allergy Institute at Mount Sinai, New York, NY, United States; 8 Asthma and Allergy Center, Lewisville and Flower Mound, Dallas, TX, United States; 9 Division of Allergy and Immunology, University of Texas Southwestern Medical Center, Dallas, TX, United States; 10 Allergy and Asthma Center, McLean, VA, United States; 11 University of Rochester, Rochester, NY, United States

**Keywords:** allergy and immunology, allergy nurses, burnout, FANA, Mini Z, nursing workforce, stress, wellness

## Abstract

**Introduction:**

Burnout among nurses remains a pervasive workforce issue in the United States, with limited data on allergy nursing. This study aimed to estimate the prevalence of burnout among U.S. allergy nurses and identify factors associated with burnout.

**Methods:**

A descriptive correlational study was conducted using the Mini Z survey and a demographic questionnaire. Responses were dichotomized into “with burnout” and “without burnout” based on validated thresholds. Descriptive statistics, Spearman's Rho correlations, and binary logistic regression were used to examine associations between burnout and workplace factors.

**Results:**

Of 241 responses, 201 met inclusion criteria. Burnout prevalence was 34% (*n* = 69). Burnout was positively correlated with job stress and work area chaos, and negatively correlated with job satisfaction, workload control, documentation time satisfaction, and professional values alignment. In the final logistic regression model, higher work area chaos (OR = 2.66; 95% CI, 1.19–5.95; *P* < 0.05) and higher job stress (OR = 4.60; 95% CI, 2.14–9.87; *P* < 0.05) were associated with increased odds of burnout, while alignment of professional values was protective (OR=0.38; 95% CI, 0.18–0.83; *P* < 0.05).

**Conclusion:**

Burnout in allergy nursing was common and associated with factors such as work environment and nurse-leadership value-alignment. Interventions that reduce practice chaos, increase workload control, streamline documentation, and strengthen alignment between nurses’ professional values and leadership may mitigate burnout. These findings can inform targeted organizational strategies, aid in the selection of existing tools, and guide the development of evidence-based interventions to reduce burnout in allergy nursing practice.

## 1 Introduction

Burnout among healthcare professionals has emerged as a pervasive and escalating crisis in the United States (U.S.), impacting clinicians across diverse practice settings and disciplines. In 2019, the World Health Organization (1) officially recognized burnout as an “occupational phenomenon,” defining it as “a long-term, unresolved, job-related stress leading to exhaustion, cynicism, detachment from job responsibilities, and lacking a sense of personal accomplishment” (2). Recent findings highlight the severity of this issue. A 2024 American Medical Association (AMA) report revealed that nearly half of physicians (48%) reported experiencing burnout (3). Similar to the AMA's findings, research by the American Nurses Association (ANA) found that among 12,581 surveyed nurses, 57% reported feeling “exhausted,” 43% identified as “burned out,” and only 20% felt valued in their roles (4). Furthermore, high levels of stress and burnout had a significant impact on nurses younger than 35 (5).

With 4.3 million licensed registered nurses (RNs) and approximately 3.5 million actively practicing RNs in the U.S., nurses represent the largest segment of the national healthcare workforce (6). The significant gap between licensed and actively practicing RNs points to a troubling trend of professional attrition, primarily attributed to burnout, and further intensified by the COVID-19 pandemic and the persistent staffing shortages that have followed (4, 7). High levels of burnout among both nurses and physicians are contributing to workforce depletion, which compounds the burden on remaining clinicians, increasing their stress and threatening the overall stability of the healthcare delivery system.

While much of the existing research on nursing burnout has focused on hospital-based nurses, emerging data show that ambulatory care nurses face similar vulnerability to burnout (8, 9). This vulnerability stems from factors such as work-life imbalance, limited organizational support, and persistent job-related stressors, many of which were exacerbated by the COVID-19 pandemic (10). According to a systematic review by Lieneck et al., substantial burnout persists among nurses working in ambulatory settings, even as the COVID-19 pandemic subsides (10).

Among ambulatory nurses, it is particularly important to understand the impact of burnout on allergy nurses given the rising prevalence of allergic diseases. Allergic diseases are a growing public health concern, affecting at least 30% of the population and nearly 80% of families (11). Given the anticipated need for more allergy nurses, understanding the prevalence and drivers of burnout are essential for retaining current nurses and attracting future nurses in this sub-specialty. Currently, the nurses in this growing sub-specialty are understudied in the nursing literature, and allergy nursing is not formally recognized by the American Nurses Association (ANA) and its subsidiary the American Nurses Credentialing Center (ANCC). Unlike other nursing specialties such as oncology, intensive care, forensic mental health nurses, or emergency nursing (12), where burnout rates have been extensively studied, there is a very limited information on burnout among allergy nurses. This represents a gap in the literature and limits the ability to design effective strategies to support the allergy nursing workforce.

Nurses in this sub-specialty typically care for patients with chronic allergic conditions, including food allergies, allergic rhinitis, and atopic dermatitis. The scope of practice for allergy nurses includes direct and indirect patient care, health education, research, interdisciplinary care coordination, and administrative tasks. In addition to the general stressors faced by ambulatory care nurses, allergy nurses may encounter unique challenges such as high patient volumes, burdensome administrative responsibilities related to prior authorizations for biologic therapies, insufficient staffing, and inadequate technological infrastructure. These factors may contribute to unrealistic workload expectations and heightened risk of burnout.

To date, limited published studies have specifically examined the size of the allergy nursing workforce or the prevalence of burnout within this group. This limited information is a barrier to designing effective strategies to support allergy nurses and address their unique needs. It is imperative to develop a deeper understanding of the allergy nursing workforce and to examine the prevalence of burnout to better identify and address the needs of nurses in this sub-specialty. Additionally, addressing burnout among allergy nurses is not only crucial for workforce retention but also for maintaining high standards of chronic disease management and patient care outcomes. Therefore, the objectives of this study were to understand (1) the demographics of the allergy nursing workforce in the U.S., (2) allergy nurses’ burnout prevalence, and (3) factors associated with burnout among allergy nurses.

## 2 Methods

### 2.1 Study design

A descriptive-correlational, cross-sectional study was conducted using the Mini Z survey (13) and a demographic questionnaire to understand allergy nursing workforce demographics, burnout prevalence, and factors associated with burnout. The Mini-Z survey was selected as it is an extensively validated tool to assess burnout and its drivers among various health care professionals. Self-reported burnout, measured using Mini-Z item 3, was the primary outcome variable. This item had five statements to select from when asked to use participants’ own definition of “burnout”: (a) I enjoy my work. I have no symptoms of burnout; (b) I am under stress, and don’t always have as much energy as I did, but I don’t feel burned out; (c) I am definitely burning out and have one or more symptoms of burnout, e.g., emotional exhaustion; (d) The symptoms of burnout that I’m experiencing won’t go away. I think about work frustrations a lot; (e) I feel completely burned out. I am at the point where I may need to seek help. Burnout categories were scored according to official scoring instructions from the Institute for Professional Worklife, with responses to statements 3a–3b classified as “without burnout” and responses to statements 3c–3e classified as “with burnout” (Supplementary Material S1). This study used a feasibility-based convenience sample drawn from national professional organizations, allergy practices and social networks.

This study was approved by the institutional review board of Molloy University. Participants were required to provide informed consent electronically first before accessing the online questionnaire.

### 2.2 Instrumentation

The Mini-Z survey is a valid and reliable instrument with reported Cronbach's Alpha between 0.7 and 0.8 and has been used previously among multiple nurse populations to assess burnout (14). Permission to use the tool, and scoring instructions were obtained from The Institute for Professional Worklife (13). The Mini Z instrument (Supplementary Material S1) is designed to evaluate workplace climate and burnout risk. In addition to burnout, the scale captures multiple drivers including perceived job stress, job satisfaction, control over workload, teamwork quality, alignment with organizational values, and burden associated with electronic medical record (EMR) documentation (13).

The electronic survey was distributed to nurses through multiple channels, including nursing-focused social media groups, researchers’ email contacts, allergy practices, and healthcare organizations where nurses are employed or volunteer. Recruitment materials directed participants to the secured survey link. Screening questions were used to assess participant eligibility by confirming U.S. residency and valid nursing credentials. Participants who met these criteria were prompted to proceed with the demographic questionnaire, followed by items from the Mini Z survey. The participants who did not meet the criteria could not proceed further. Skip logic was applied to advance survey questions based on participant responses limiting to one submission per person.

Demographic questions in the questionnaire included data about participant age, gender, race, education level (Diploma, Associate, Baccalaureate, Master's, or Doctorate), state of practice, total years of nursing practice, and years of clinical practice within the allergy sub-specialty. Additionally, questions were included related to role, hours worked per week, area of focus (adult or pediatric), practice setting (small private practice, practice affiliated with a large academic center, government funded institution, etc.), sub-specialty/practice environment, certifications, and memberships in professional organizations. Two questions related to self-reported contributing factors to burnout and most helpful strategies in strengthening well-being were included. Participants were also given the opportunity to provide open-ended feedback in a comment section. All close-ended items were required, and open ended items remained optional.

### 2.3 Study procedures and data collection

Nurses were recruited from relevant professional organizations including the American Academy of Allergy, Asthma & Immunology (AAAAI), Food Allergy Nursing Association (FANA), American Society of Allergy Nurses (ASAN), National Coalition of Ethnic Minority Nurses Associations (NCEMNA), Philippine Nurses Association of America (PNAA), American Academy of Ambulatory Care Nursing (AAACN), Society of Nurse Scientists, Innovators, Entrepreneurs & Leaders (SONSIEL), Orthodox Jewish Nurses Association (OJNA), The Texas Allergy, Asthma and Immunology Society (TAAIS), authors institutions and allergists practices, social media network groups, and email listservs. Recruitment was conducted by distributing an electronic flyer containing a survey link, including an electronic consent, through these organizations and networks. The survey was administered electronically through a secure web-based form and required approximately 7–10 min to complete. Data were collection between November 18, 2024 and December 30, 2024. No incentives were provided for participation. Upon submission, participants had the option to voluntarily provide their contact information for potential inclusion in future studies. No identifying information was collected and all responses were treated as confidential. Because the survey was disseminated through open online channels where the number of individuals exposed to recruitment materials could not be determined, the response rate was calculated based on competed surveys received.

### 2.4 Sample size

Using the common rule of thumb for regression analyses with six or more predictors, a minimum of 10 participants per predictor variable is recommended (15). Additionally, published Mini-Z studies demonstrate use across a wide range of sample sizes, from large multisite cohorts to smaller validation studies (∼120 participants) (10, 14, 16); therefore, a target sample of ∼200 participants was judged adequate and consistent with prior Mini-Z burnout research.

### 2.5 Eligibility criteria

Inclusion criteria were operationalized as follows: licensed nurses, who are able to read and respond in English, over the age of 18 years old, practicing within the sub-specialty of allergy in the U.S. Individuals under 18 years old, non-U.S. residents and nurse practitioners functioning in the provider capacity were excluded from the study.

### 2.6 Statistical analyses

The survey data was downloaded to a Microsoft Excell sheet and was cleaned and coded by two research team members (LM and OK) to ensure accuracy. The de-identified dataset was then uploaded to IBM SPSS Version 28.0 for analysis. Statistical tests used were aligned to study aims, and it included descriptive statistics, correlation analysis, logistic regression, and cross-tabulations to examine demographic distributions, burnout prevalence, and its associated factors. To determine factors which predicted the probability of burnout using the burnout drivers measured in the Mini-Z survey, we used logistic regression, which predicts a binary outcome given one or more independent variables. To determine the relationship between burnout and the burnout drivers measured in the Mini-Z survey, we used Spearman correlation. Assumptions for non-parametric correlations and binary logistic regression analysis were met as dependent variable was discrete and dichotomous.

## 3 Results

### 3.1 Demographics

**Figure 1:**
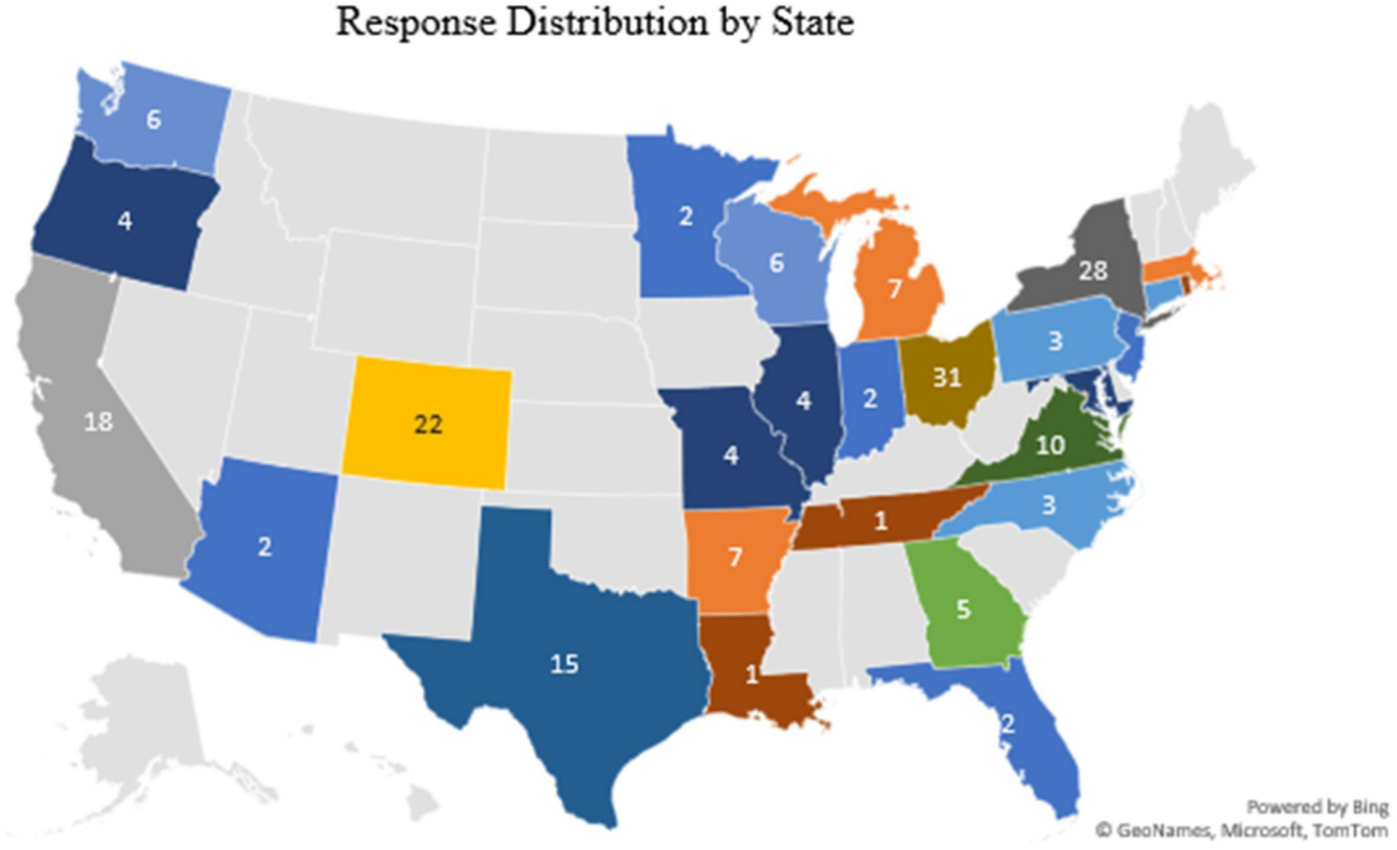
Geographic distribution of responses.

**Figure 2:**
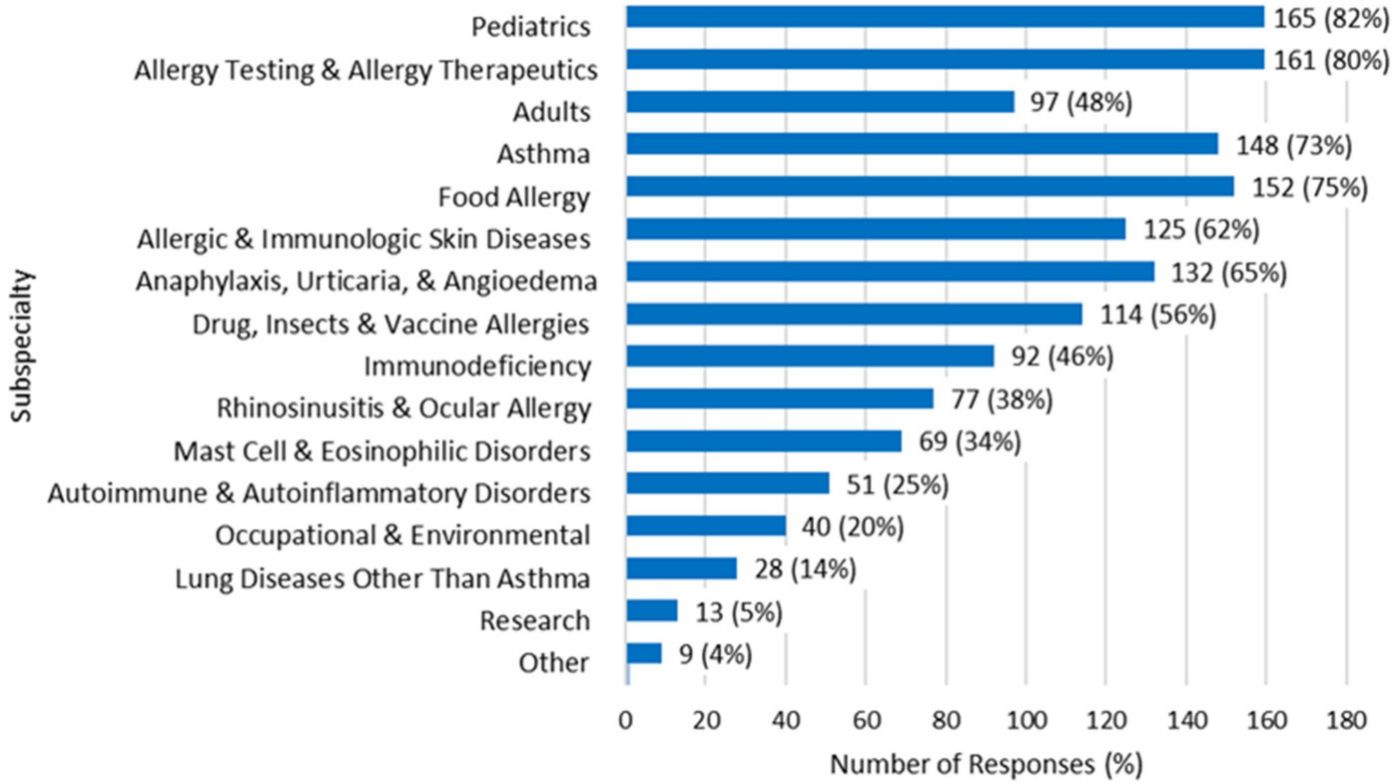
Sub-specialty or areas of practice. *N* = 201. Other: Public health education, allergy shots, injection rooms, high risk multidisciplinary clinic, immunotherapy, administration, primary care, treatment center.

Of the 241 responses received, 201 met the inclusion criteria. Of 201 participants, 34% (*n* = 69) reported experiencing burnout. Descriptive analysis indicated that the majority of respondents had a Baccalaureate Degree (65%, *n* = 130), identified as Caucasian (79%, *n* = 158), identified as women (94%, *n* = 188), had eleven or more years of experience (70%, *n* = 140), worked in an outpatient setting (75%, *n* = 151), and did not belong to professional organizations 58% (*n* = 116) or held professional certifications 59% (*n* = 118) (Table 1). The states with the highest number of responses were New York (*n* = 28), followed by Colorado (*n* = 22), California (*n* = 18), and Texas (*n* = 15) (Figure 1). Nurses across several sub-specialty areas of practice were included (Figure 2), with the most representation in pediatrics (*n* = 165, 82%).

**Table 1:** Demographic data.

Demographic	*N* (%)
Educational Credentials	
RN Diploma^a^	7 (3%)
RN Associate Degree	20 (10%)
RN Baccalaureate Degree	130 (65%)
RN Masters, not APRN	29 (14%)
Doctoral Degree, not APRN	3 (2%)
LPN/LVN	12 (6%)
Gender	
Female	188 (94)
Male	9 (4%)
Prefer not to disclose	4 (2%)
Race/Ethnicity	
White	158 (79%)
Hispanic or Latino	9 (4.5%)
Black or African American	11 (5%)
Asian	12 (6%)
Native Hawaiian or Other Pacific Islander	2 (1%)
American Indian or Alaskan Native	1 (0.5%)
Other	8 (4%)
Nursing Experience	
<5 yrs	20 (10%)
5–10 yrs	41 (20%)
11–20 Yrs	62 (31%)
>20 yrs	78 (39%)
Primary work setting	
Hospital owned practice	38 (19%)
Outpatient, Academic center	52 (26%)
Outpatient, Multispecialty Clinic	73 (36%)
Outpatient/Ambulatory Immunology Allergy	20 (10%)
Outpatient, Community	6 (3%)
Other	12 (6%)
Membership in Professional Organization	
No	116 (58%)
Yes*	85 (42%)
Professional Certifications	
No	118 (59%)
Yes**	83 (41%)
AE-C	16 (19%)
AMB-BC™	19 (23%)
CPN	32 (38%)
Other	28 (33%)

*N* = 201.

Abbreviations: RN, registered nurse; APRN, advanced practice registered nurse; LPN, licensed practical nurse; LVN, licensed vocational nurse.

* Most commonly listed: ANA, American nurse association; AAAAI, American academy of allergy, asthma and immunology; ASAN, American society of allergy nurses; FANA, food allergy nursing association; AAACN, ACAAI, American college of allergy, asthma & immunology; PNAA, Philippine nurses association of America; American academy of ambulatory care nursing.

** Certifications types: AE-C (Asthma Educator); AMB-BC™ (Certified Ambulatory Nurse); CPN (Certified Pediatric Nurse).

^a^ Nursing diploma degree is one of the pathways to licensure in the United States (HRSA, 2028).

### 3.2 Burnout prevalence

Among participants who reported burnout (*n* = 69), prevalence was higher among allergy nurses working more than 40 hours per week (58%), with low job satisfaction (49%), experiencing job stress (62%), less workload control (66%), lower satisfaction with documentation time (68%), working in a chaotic atmosphere (63%), and those whose professional values did not align with their leadership (51%) (Table 2).

**Table 2:** Burnout prevalence.

Characteristic	All respondents	Without burnout	With burnout
	*n* = 201	*n* = 132	*n* = 69 (34%)
Gender			
Female	188	124	64 (34%)
Male	9	6	3 (33%)
Prefer not to disclose	4	2	2 (50%)
Credentials			
RN Diploma	7	5	2 (29%)
RN Associate Degree	20	11	9 (45%)
RN Baccalaureate Degree	130	87	43 (33%)
RN Masters, not APRN	29	21	8 (28%)
Doctoral Degree, not APRN	3	1	2 (67%)
LPN/LVN	12	7	5 (42%)
Work Hours			
<20	8	7	1 (13%)
21–25	25	16	9 (36%)
26–30	17	13	4 (23%)
31–35	28	22	6 (21%)
36–40	80	56	24 (30%)
>40	43	18	25 (58%)
Professional Membership			
No	116	83	33 (28%)
Yes	85	49	36 (42%)
Professional Certifications			
No	118	76	42 (36%)
Yes	83	56	27 (32%)
Job Satisfaction			
No	59	30	29 (49%)
Yes	142	102	40 (28%)
Job Stress			
No	133	106	27 (24%)
Yes	68	26	42 (62%)
Workload Control			
No	50	17	33 (66%)
Yes	151	115	36 (24%)
Documentation Time Satisfaction			
No	34	11	23 (68%)
Yes	167	121	46 (27%)
Work Chaos			
No	139	109	30 (22%)
Yes	62	23	39 (63%)
Professional Values Alignment			
No	77	38	39 (51%)
Yes	124	94	30 (25%)
Care Team Work Efficiency			
No	11	6	5 (45%)
Yes	190	126	64 (34%)
EMR Documentation at home			
No	187	124	63 (34%)
Yes	14	8	6 (43%)
Gender Discrimination			
No	193	130	63 (33%)
Yes	8	2	6 (75%)
Racial Discrimination			
No	198	131	67 (34%)
Yes	3	1	2 (67%)
Religious Discrimination			
No	201	132	69 (34%)
Yes	0	0	0 (0%)

*n* = 201 for the burnout survey.

### 3.3 Predictors of burnout

Spearman Rho correlation analysis revealed that burnout was positively associated with job stress (*r* = 0.413, *P* < 0.05) and work area chaos (*r* = 0.402, *P* < 0.05), and negatively associated with job satisfaction (*r* = −0.201, *P* < 0.05), workload control (*r* =−0.384, *P* < 0.05), documentation time satisfaction (*r* = −0.317, *P* < 0.05), and professional values alignment (*r* = −0.271, *P* < 0.05) (Table 3).

**Table 3:** Correlations analysis.

Spearman's Correlation	Job Satisfaction	Job Stress	Burnout	Workload Control	Documentation Time Satisfaction	Work Area Chaos	Professional Values Alignment
Job Satisfaction	1						
Job Stress	−0.116	1					
Burnout	−.201**	.413**	1				
Workload Control	0.008	−.367**	−.384**	1			
Documentation Time Satisfaction	−0.029	−.210**	−.317**	.477**	1		
Work Area Chaos	−0.137	.297**	.402**	−.388**	−.474**	1	
Professional Values Alignment	.301**	−0.021	−.271**	.186**	.190**	−.205**	1

** Correlation is significant at the 0.01 level (2-tailed).

This study employed binary logistic regression to examine factors contributing to the probability of burnout. Six independent variables that previously demonstrated statistically significant correlations with burnout were entered in the regression model stepwise in the following order: job satisfaction, workload control, work area chaos, professional values alignment, job stress, documentation time satisfaction. The best logistic regression model was significant, *χ*²(6, *N* = 201) = 71.628, *P* < 0.001, indicating a strong relationship between burnout and work area chaos (OR = 2.660, 95% CI, 1.190–5.950, *P* < 0.05), professional value alignment (OR = 0.383, 95% CI, 0.176–0.834, *P* < 0.05), and job stress (OR = 4.596, 95% CI, 2.140–9.870, *P* < 0.05). Participants who reported higher levels of work area chaos were 2.66 times more likely to experience burnout. Conversely, those with professional values alignment with their leadership were less likely to experience burnout (OR = 0.383). Additionally, higher reported job stress significantly increased the likelihood of burnout by approximately 4.6 times. While job satisfaction, workload control, and documentation time satisfaction demonstrated significant associations with burnout in the correlation analysis, they did not emerge as significant predictors of burnout in the regression model.

### 3.4 Burnout contributors

**Figure 3:**
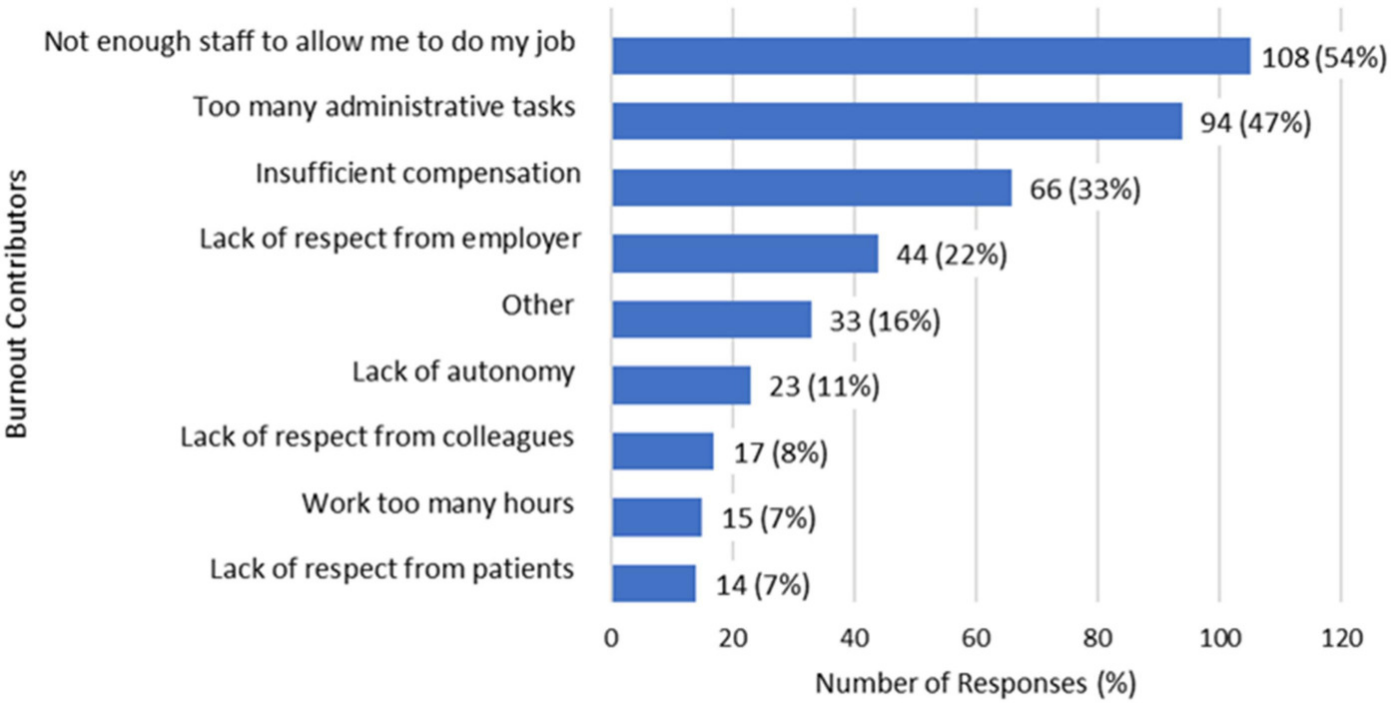
Most common contributors to burnout (*N* = 201).

Upon frequency analysis of the first additional question, the most common self-reported contributor to burnout was a lack of support-staff necessary for job performance (54%, *n* = 108), followed by a preponderance of administrative tasks (47%, *n* = 94) and then insufficient compensation (33%, *n* = 66) and lastly, lack of respect from employers (22%, *n* = 44) (Figure 3).

### 3.5 Well-being contributors

**Figure 4:**
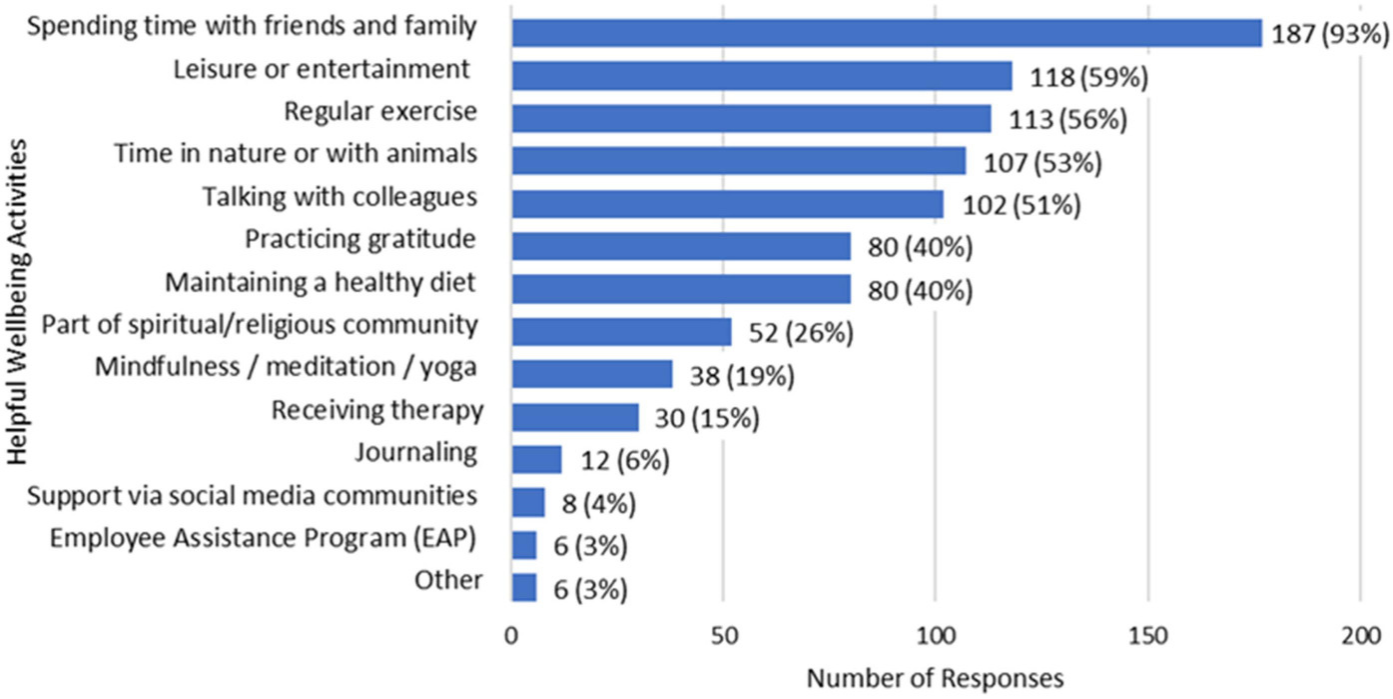
Most helpful activities in strengthening well-being (*N* = 201). Other: Setting boundaries, restricting work email access while on vacation, reading, crafts, volunteer groups, stepping out of practice, consideration of leaving the nursing profession*.*

Upon frequency analysis of the second additional question, spending time with friends and family (87%, *n* = 187) was the most helpful self-reported activity in strengthening a sense of well-being among survey participants, followed by engaging in leisure or entertainment activities (59%, *n* = 118) and regular exercise (56%, *n* = 113). Other helpful activities included time with nature/animals, conversations with colleagues, and practicing gratitude among others as shown in Figure 4.

### 3.6 Textual analysis

Ninety participants provided comments in the survey, which were content analyzed and categorized using the *Stanford Model of Occupational Well-being*,™ formerly known as the *Stanford Model of Professional Fulfillment* framework (17). This model consists of three domains: culture of wellness, efficiency of practice, and personal resilience.

#### 3.6.1 Culture of wellness

Survey participants’ comments highlighted a lack of understanding and engagement from leadership regarding the unique challenges of allergy nursing, including understaffing, space constraints, and unrealistic workload expectations. Nurses emphasized the need for clear communication, respect, and collaboration among staff and physicians to improve morale and job satisfaction. Recognition of nursing expertise, alongside efforts to create safe and equitable work environments, was repeatedly identified as critical. A shift away from profit-driven priorities toward a more patient-centered and staff-centered culture was desired to enhance overall wellbeing and reduce burnout.

#### 3.6.2 Efficiency of practice

Respondents identified insufficient staffing, burdensome administrative tasks, and inadequate technological systems as major contributors to stress. High patient volumes, coupled with a lack of resources, such as triage nurses, proper equipment, and training, exacerbated workload challenges. Efficient task delegation, streamlined processes, and better support from leadership were recommended to improve practice operations. Respondents reported that addressing these inefficiencies are essential for maintaining patient safety, reducing errors, and enabling nurses to focus on core clinical responsibilities.

#### 3.6.3 Personal resilience

Nurses frequently cited the need for work-life balance and personal coping strategies to manage the demands of their roles. Many relied on mindfulness, yoga, exercise, or disconnecting from work to mitigate stress. Financial concerns, including inadequate compensation and the necessity of secondary employment, were noted as stressors that could be alleviated through better pay and benefits. Additionally, the ability to work remotely or have flexible schedules was highlighted as a way to improve resilience. Nurses also expressed the importance of professional respect, meaningful contributions, and maintaining boundaries between their personal and professional identities to sustain long-term fulfillment and reduce burnout.

## 4 Discussion

### 4.1 Allergy nursing workforce

There is limited prior research on burnout among allergy nurses. This descriptive-correlational study examined the demographic characteristics of allergy nurses in the U.S., their burnout, and the factors associated with it. The demographic distribution of respondents, particularly regarding race and gender, closely mirrors the U.S. national nursing workforce, and the geographic distribution of responses aligns with the national distribution of active RN licenses by state, with notable response density from California, Texas, Colorado, Ohio, New York, and Virginia (18, 19). The majority of respondents who worked more than 40 hours per week (58%) reported burnout which aligns with prior study findings supporting a strong positive relationship between working long hours and adverse nurse and patient outcomes (20, 21, 22).

### 4.2 Prevalence and predictors of burnout

Overall, the outcomes of this study are encouraging as burnout rates were lower in comparison to nurses working in in-patient settings, such as critical care (44%) (23). When compared with allergy/immunology physician populations, as both share a similar work environment, several parallels emerged. While allergy nurse burnout was slightly lower (34%) than that of practicing allergists (39%), this study revealed statistically significant findings in job stress, documentation time, value alignment, workload control, and work environment, mirroring patterns reported among allergists (24). Furthermore, as with physicians, burnout poses a significant risk to nurses’ well-being, impacting job satisfaction and retention (25). Notably, job satisfaction among ambulatory nurses dropped from 89% in 2018 to 80% in 2022 (8), a trend that may compound burnout among already short-staffed nursing teams. Nurses in this study and allergists in prior studies have cited burdensome administrative and documentation tasks as major contributors to burnout (26, 27). These similarities highlight that burnout in allergy care is a systemic issue affecting multiple members of the clinical team.

Recent data show that physician burnout and depression rates declined in 2024 compared to 2022, likely due to positive coping strategies such as regular exercise, adequate sleep, music, and quality time with family and friends (28). Although these improvements were observed among physicians, similar progress may be achievable within the allergy nursing workforce, given that nurses and physicians operate within the same interdisciplinary teams, care for the same patient population, and navigate similar practice environments. However, interventions may require adaptation to reflect the distinct roles and responsibilities of nurses in allergy care.

The study findings indicated that burnout among allergy nurses is a multifactorial phenomenon influenced by both work environment and interpersonal factors. Associations were found between burnout and chaotic environment, professional value alignment, and job stress. Nurses who reported higher job stress were 4.6 times more likely to experience burnout. This is an important finding that highlights the impact of workplace stress as cited in prior studies among U.S. nurses, using national survey data (29). Participants who reported higher levels of chaos in their work environment were 2.66 times more likely to experience burnout. Chaotic work environment further exacerbates risk of burnout, as demonstrated by Chiminelli-Tomás et al. (30), who found that disorganized practice settings correlate with increased emotional exhaustion, while a favorable professional practice environment relates to lower burnout symptoms. Conversely, alignment between nurses’ professional values and organizational culture serves as a protective factor, reducing burnout prevalence (31). Nurses who felt their professional values aligned with their leadership and organization were less likely to experience burnout. Additional contributors to nurse well-being included job satisfaction, workload control, and satisfaction with documentation time. Studies by Diehl et al. (32), and Wells (33) emphasize that supportive environments and manageable workloads buffer against burnout, while excessive documentation demands are linked to clinician fatigue and disengagement. These findings highlight the importance of fostering structured, communicative, and value-driven workplaces to mitigate burnout and promote sustainable nursing practice.

In addition to documentation demands, inadequate staffing emerged as a strong contributor to burnout among allergy nurses. According to the National Sample Survey of Registered Nurses (NSSRN), 50% of nurses who left the profession in 2022 did so due to burnout, and 39% cited inadequate staffing as a contributing factor (8). Staffing shortages intensified workload and limited opportunities for rest, peer collaboration, and engagement in professional development activities (29, 32). In allergy practices, the specialized demands of biologic therapy administration require enhanced clinical oversight and coordination. The introduction of newly approved biologic therapies for allergic conditions has increased the need for nursing resources both for clinical care delivery and administrative coordination, emphasizing the urgency of targeted interventions to address workforce strain in allergy settings (25). Customizing wellness initiatives to reflect the unique operational and emotional demands of allergy care may prove essential to sustaining the workforce and reducing attrition.

### 4.3 Promoting efficiency of practice, culture of wellness, and personal resilience

The analysis showed that job stress, work area chaos, and professional values alignment were significant predictors of burnout. To complement this study's quantitative findings, we examined nurses’ open-ended comments through the lens of the Stanford Model of Occupational Well-being™, which emphasizes three interrelated domains: efficiency of practice, culture of wellness, and personal resilience. These domains provide a framework for addressing the organizational factors identified in this study. Across these domains, nurses described strategies that correspond to predictors: reducing chaos through workflow redesign, lowering stress via workload control and scheduling, and strengthening value alignment through leadership engagement. Examples include streamlined EMR, which some researchers suggest fosters nurses’ well-being at work (34), adequate staffing, balanced schedules, improved communication, recognition of nursing expertise, and access to wellness resources. This study's findings, supported by previous research, suggest that optimizing practice efficiency can alleviate workload-related stress and burnout among clinicians (25, 27). Leadership engagement is equally important, as studies show that organizations fostering a culture of wellness and aligning organizational values with individual purpose experience lower burnout rates (9, 17, 26, 35). Practices such as regular staff check-ins, transparent communication, and shared decision-making reinforce value alignment and promote psychological safety (36). The following sections explore each domain in greater detail, integrating nurse perspectives with evidence-based recommendations.

#### 4.3.1 Culture of wellness

The study's results highlight value alignment as a protective factor against burnout, making culture of wellness an important priority. Nurses emphasized clear communication, mutual respect, and collaboration to improve morale. Their recommendations directly address the alignment gap identified in the regression analysis and complement the study's findings by emphasizing leadership behaviors aligning with organizational and professional values. Studies suggest a strong link between a positive organizational culture and burnout symptoms (35, 37–40). As clinician well-being remains a national priority, recognized within the Quintuple Aim (39) and the National Academies of Medicine's Plan for Health Workforce Well-Being (40), ANA has taken initiatives to address it. Credentialed programs such as the ANA Well-Being Excellence™ initiative (41) offer evidence-based standards to operationalize these changes. For allergy practices, this may include regular staff check-ins, policy adjustments to reduce stressors, and recognition programs that honor nursing expertise and contributions to patient care, aligning with both patient and staff values. As Gallup data (42) show continued declines in workforce well-being, nurse leaders must model wellness practices and foster inclusive support networks. Prioritizing culture change is both a moral imperative and a strategic investment in workforce sustainability.

#### 4.3.2 Efficiency of practice

Work area chaos was a significant predictor of burnout in this study, supporting the need for interventions that improve practice efficiency. Nurses identified burdensome documentation, inadequate staffing, and lack of triage support as stressors that increase cognitive load, which are factors contributing to chaotic environments. Strategies such as automation tools and digital platforms can streamline documentation and reduce time spent on non-clinical tasks, while additional staffing and new models of care can redistribute administrative responsibilities (28, 43). Efficient task delegation, resource allocation, and leadership responsiveness were repeatedly recommended to reduce frustration and improve practice operations. Organizational interventions that promote emotional recovery and peer connection have shown promise in improving clinician well-being (44). For instance, a proactive peer-to-peer support program implemented at a pediatric medical center demonstrated high engagement and perceived value among frontline staff (45). Another example is Schwartz Rounds®, a compassion practice program enhancing staff caring and teamwork (46). Rest breaks have been found to be effective in decreasing professional burnout among registered nurses (47). These models may offer scalable approaches to mitigating burnout in specialty settings like allergy care. We encourage employers to prioritize workflow redesign, technology upgrades, and team-based support systems to sustain workforce performance and patient safety.

#### 4.3.3 Personal resilience

Although systemic changes are essential, the findings and open-ended responses indicate that personal resilience remains an important buffer against stress. Nurses described coping strategies such as mindfulness, exercise, and disconnecting from work approaches that complement organizational efforts to reduce chaos and stress. These findings align with broader literature showing that self-care and emotional regulation are protective against burnout (24, 48–50). Financial stressors and secondary employment, reported by participants, highlight the need for adequate compensation and benefits (29). Flexible schedules and remote work options were also emphasized, aligning with evidence that workplace commitment and team cohesion buffer workload effects (32). Structured resilience programs, such as team-based interventions (51), and strategies for self-awareness and boundary-setting (52) can further strengthen resilience and sustain well-being.

## 5 Strengths and limitations

This study provides a novel investigation into burnout among allergy nurses addressing a gap in the literature where most research focuses on inpatient settings or pandemic-related burnout. Strengths include the study's focus on a specialized workforce and its use of a validated instrument to examine both prevalence and associated factors. By identifying job stress, work area chaos, and professional values alignment as significant predictors, these findings offer actionable insights for improving workforce sustainability and patient care. The results highlight the need for targeted interventions that address organizational and individual factors contributing to burnout. Given that these nurses provide essential care to patients with chronic allergic conditions, improving their well-being is vital for both workforce sustainability, quality care, and patient outcomes. This research has the potential to guide meaningful solutions for mitigating burnout and enhancing clinician support as part of the larger interprofessional care team.

The study's cross-sectional design is reflective of data at one point in time and does not allow evaluation of longitudinal relationships, which limits understanding of how these factors relate over time. The sample was predominantly white and female, despite efforts to recruit diverse nurses from the National Coalition of Ethnic Minority Nurses Associations (NCEMNA), the Philippine Nurses Association of America (PNAA), and the DEI committee of the AAAAI, and findings may not be generalizable to other specialties, demographic groups, or international contexts. Although recruitment was conducted across multiple organizations and practice settings, the feasibility-based convenience sampling approach introduces the potential for self-selection and response bias, as individuals with greater interest in a topic or concern about workplace well-being may have been more likely to participate. Furthermore, generalizability may be restricted beyond the specific institutional, professional, and geographic networks accessed in this study. However, the broad geographic distribution of responses across U.S. regions supports the generalizability of findings across major nursing regions (18, 19). Despite these several limitations, this study offers valuable preliminary insights into the allergy nursing workforce. Future research employing larger, more heterogeneous samples, probability-based sampling strategies, and objective workload indicators would be ideal for deepening researchers’ understanding of burnout within allergy nursing.

## 6 Recommendations

The recommendations stem directly from the findings. At the institutional level, organizations should prioritize interventions that reduce practice chaos and job stress, such as optimizing EMR systems, increasing support staff, and implementing policies that promote balanced workloads (9, 27, 49). Leadership engagement strategies that strengthen value alignment, such as regular staff check-ins, transparent communication, and recognition programs that are essential for reducing burnout risk (35, 36). On an individual level, resilience-building and self-care training should be integrated into professional development programs to empower clinicians to manage stress effectively (24, 48). Resources such as the ANA and AAAAI wellness toolkits (53, 54) and emerging Artificial Intelligence-enabled tools (55) can support these efforts. Additional resources are outlined in Table 4. Specialty organizations like AAAAI and FANA can spearhead tailored wellness initiatives to address the unique demands of allergy care, ultimately improving job satisfaction, patient safety, and workforce sustainability. As researchers continue to observe persistently high rates of burnout and mental health challenges among healthcare professionals in both low and high resource countries (56), future studies should include nurses and allied health professionals internationally to provide a broader perspective on burnout within allergy care.

**Table 4:** Selected wellness resources.

Source	Description
American Nurses Association (57)	Comprehensive tools for mental health support, including virtual and in-person assistance, video and podcast skills training, and mood improvement strategies. Examples: mHealth app Moodfit: Mood journals, breathing exercises, & more. Vitalize Care app (iPhone). Vitalize Care app (Android). Telehealth (via phone) Toll Free—(833)327-0262 or text your first name to Happy at (858)367-3001 to talk confidentially to compassionate listeners anytime you need about wellness, recovery, and resilience. Locate a mental health professional The Nurses’ Guide to Mental Health Support Services can help you better understand what support systems and services are available to you and how to locate them. Podcasts 9-part podcast mini-series: how to implement the gratitude toolkit into your daily life. “A Nursing State of Mind”: two veteran nurses discuss coping mechanisms with practical ideas to renew our energy, confidence, and passion for nursing.
American Holistic Nurses Association (58)	Offers self-care and resilience resources for nurses, including various self-care strategies and resilience-building tools.
Greater Good Science Center (59)	Accessible resources on positive psychology, gratitude, and resilience such as online courses and psychological tools for well-being.
Healthy Nurse Healthy Nation (60)	Focuses on mental health, physical activity, nutrition, rest, quality of life, and safety. It supports nurses’ overall well-being across six key areas.
PAM-Pause A Moment (61)	Offers stress management activities to recognize and manage stress, cultivate flexible thinking, and find solutions to challenges.
R³—the Renewal, Resilience and Retention of Maryland Nurses Initiative. The Johns Hopkins University School of Nursing (62)	Research-driven initiative at Johns Hopkins to support nurses which focuses on resilience-building and retention strategies for nurses.

## 7 Conclusion

In conclusion, this study suggests that burnout among allergy and immunology nurses is a complex, multifaceted issue shaped by several factors and is a serious concern that impacts both workforce sustainability and patient care. This study identifies key contributors to burnout among allergy nurses and emphasizes the need for targeted organizational strategies and personal resilience initiatives. By integrating institutional interventions, fostering personal coping strategies, and targeting specialty-specific risks, health care organizations can cultivate a culture of wellness that supports the needs of clinicians and enhances patient care outcomes. Continued monitoring and research into burnout will be essential to refine these strategies and ensure a resilient and sustainable allergy care workforce.

## Data Availability

The raw data supporting the conclusions of this article can be made available by the authors upon request.
